# Scotch Servants and Hospital Problems

**Published:** 1914-08-29

**Authors:** 


*To the Editor of* The Hospital.

### Scotch Servants and Hospital Problems.

*To the Editor of* The Hospital.

Sir,—Under the above heading in your issue of July 25 it is asserted that by the common law employers are liable to support a domestic servant for six weeks during ill-health. Am I right in supposing that there is no such law in England? L. J.

[Under the common law of England it is the primary duty of a master on taking a servant into his employ to enable him to earn the wages agreed to be paid, and if he neglects so to do he renders himself liable to an action for breach of contract. The master, however, is not liable for surgical or medical attendance and the medicine supplied to a servant who has sustained illness during the period of his service, provided that such illness has not been caused by the master's negligence and unless the surgeon or doctor has been called in by the master's orders. And where a servant has been compelled, through illness, to absent himself from his work his master will be bound to pay him his wages until the proper termination of his employment—namely, by notice or by effluxion of time. Common law, as well as statute law —namely, the Fatal Accidents Act, 1845; the Employers' Liability Act, 1880, and the Workmen's Compensation Act, 1906—provide for "accidents" to servants and employees and for the master's liability in respect thereof, but a discussion of their provisions does not, it is considered, come within the purview of, nor affect the question submitted.—Ed. The Hospital.]

